# Brachydactyly Type A3 Is More Commonly Seen in Children With Short Stature But Does Not Affect Their Height Improvement by Growth Hormone Therapy

**DOI:** 10.3389/fendo.2022.824315

**Published:** 2022-02-03

**Authors:** Huahong Wu, Yang Li, Hui Li

**Affiliations:** Department of Growth and Development, Capital Institute of Pediatrics, Beijing, China

**Keywords:** brachydactyly, short stature, children, growth hormone, height

## Abstract

**Introduction:**

To analyze the prevalence of brachydactyly type A3 (BDA3) in children with short stature and the effect on growth hormone (GH) therapy.

**Methods:**

We analyzed the medical records of pediatric patients from July 2009 to July 2021. We included children with short stature defined as their height standard deviation score (HtSDS) < -2 and normal short height as their HtSDS between -2 and -1. We calculated the prevalence of BDA3 in different groups and compared the differences in children’s characteristics and the therapeutic effect of GH therapy between the BDA3 and no BDA3 groups.

**Results:**

A total of 752 cases were included. The overall prevalence of BDA3 was 23.1%; with a female predominance (30.8% vs. 16.1%, *P* < 0.01). BDA3 was more prevalent in the short stature group (27.2%) than in the normal short stature group (16.7%) and growth hormone deficiency group (16.5%). Birth length, birth weight, HtSDS, and mid-parental height of children with BDA3 were lower than those without BDA3, but there were no significant differences. In patients with Turner syndrome and idiopathic short stature, the HtSDS of the BDA3 group was significantly lower than that of the no BDA3 group (*P* < 0.01). During four years of GH therapy, the HtSDS improvement per year in the BDA3 group were 0.79 ± 0.29, 0.50 ± 0.31, 0.20 ± 0.30, and 0.10 ± 0.22, which were not significantly different from those in the no BDA3 group. At the end of treatment, there were no significant differences in the duration of treatment and total HtSDS improvement between these two groups.

**Conclusions:**

BDA3 is more commonly seen in children with short stature with a female predominance. BDA3 occurrence is independent of the GH pathway and does not affect the therapeutic effect of GH on short stature children.

## Introduction

Brachydactyly type A3 (BDA3) is the most common hand anomaly characterized by a shortened middle phalanx of the fifth finger (1). BDA3 often occurs as an isolated malformation in Chinese children, which can be simply identified by a left-hand wrist X-ray film in pediatric clinics. The prevalence of BDA3 varies significantly among different races, with the highest prevalence in the Asian population and lowest in European and African descents. The prevalence of BDA3 ranged from 8.6%–25.6% in Japanese, 1.0%–19.5% in Native Americans, and 0%–2.1% in European and African descents (2, 3). Notably, Europeans are among the tallest populations worldwide, while Asians are among the shortest (4); thus, we can assume that the prevalence of BDA3 in different populations is inversely related to their average height. The occurrence of BDA3 is related to the disorder of cartilage ossification at the epiphysis and advanced closure of the epiphysis, which is also an essential process in height growth (5). Therefore, BDA3 may be related to height growth. However, the exact mechanism of BDA3 and its association with height growth remain unclear.

In recent years, we have observed an increased incidence of BDA3 during growth and bone age evaluation based on the left-hand wrist X-ray film in pediatric clinics. Most children visited a doctor for short stature (6). For short children with BDA3, there has been no research on whether BDA3 is associated with short stature and whether growth hormone (GH) therapy can effectively improve their height compared to those without BDA3. Therefore, we used real-world data from pediatric clinics to analyze the prevalence of BDA3 in a short stature population, the characteristic differences between children with or without BDA3, and whether GH therapy is effective for short children with BDA3. These findings can provide evidence for understanding the relationship between BDA3 and short stature and can aid in diagnosing and treating children with BDA3 in clinical practice.

## Materials and Methods

### Data Resource

Medical records were collected retrospectively from the Growth and Development Clinic of the Capital Institute of Pediatrics, Beijing, China, from July 2009 to July 2021. Considering that the physical growth and development level of children with precocious puberty or advanced development was inconsistent with their chronological age, which may affect the evaluation of children’s actual height level, we excluded children diagnosed with precocious puberty and advanced development with normal height in this study. Therefore, only complete medical records with physical measurements, a left-hand wrist X-ray film, definite diagnosis, regular follow-up, therapy information, and those with short stature or normal shorter height were included. Meanwhile, we required a left-hand wrist X-ray film that would allow precise identification of the presence of BDA3. The diagnosis of BDA3 for all patients was strictly assigned following the diagnostic criteria.

### Sample Screen and Division

**Figure 1:**
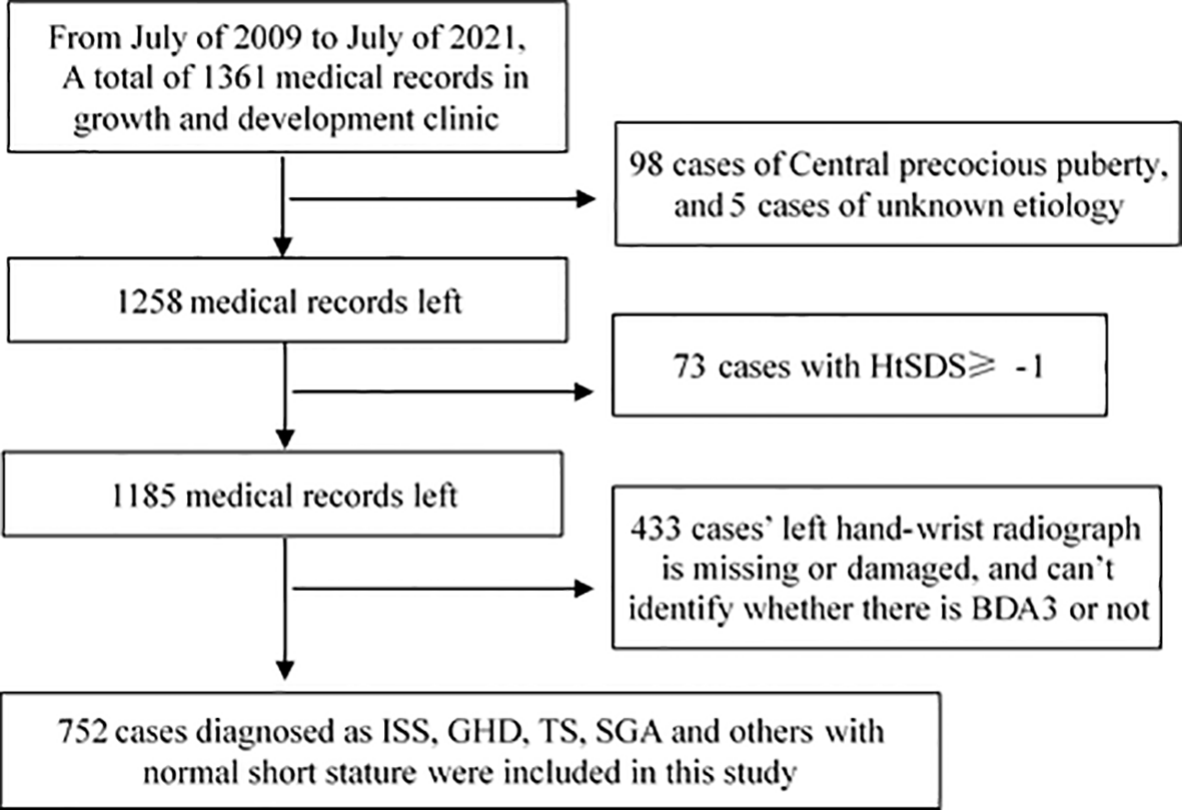
Screening procedure of survey samples.

The height and weight recorded in medical cases were measured by trained staff or nurses. We calculated the standard deviation score of patients’ height (HtSDS) and weight (WtSDS) using Chinese children’s growth references (7). In this study, an HtSDS of < -1 was defined as normal short height; therefore, cases with HtSDS of ≥ 1, usually with advanced bone age, were excluded. We divided all included cases into two groups: those with an HtSDS < -2 as the short stature group and those with an HtSDS between -2 and -1 as the normal short group. The screening procedure is shown in **Figure 1**.

### Definition of BDA3

**Figure 2:**
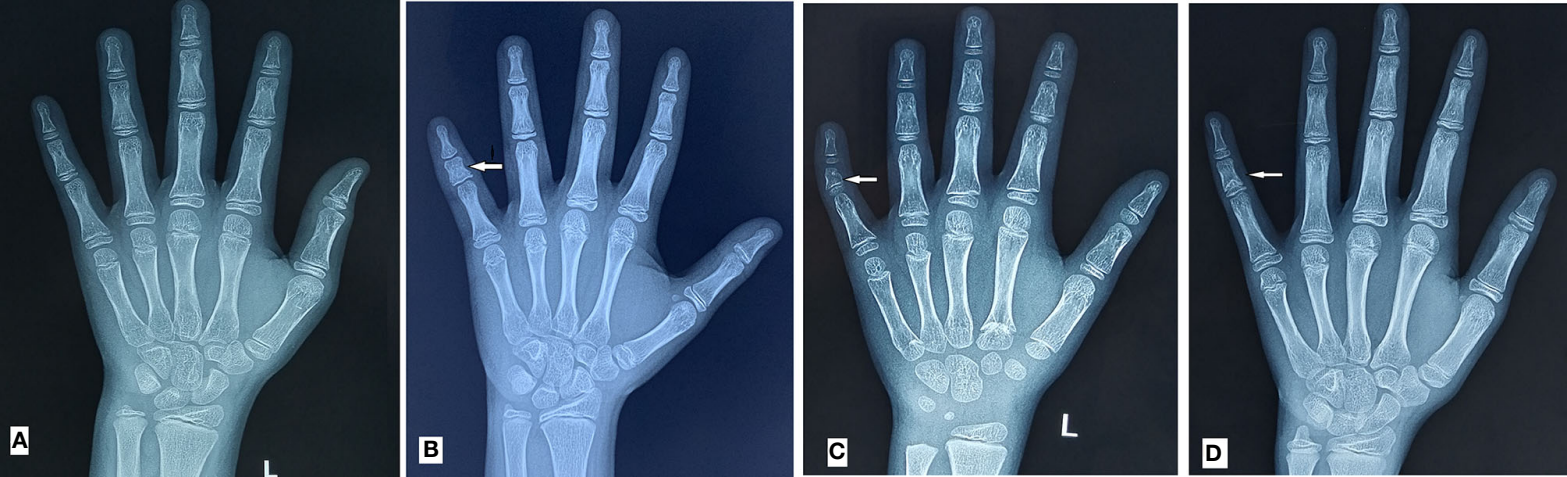
BDA3 diagnostic diagram. **(A)** is the normal left hand-wrist X-ray film; **(B)** is the middle phalanx of fifth finger shorter than half of the middle phalanx of fourth finger; **(C)** is the shorter middle phalanx of fifth finger with curved middle phalanx of fifth finger to the radial side; **(D)** is the shorter middle phalanx of fifth finger with conical epiphysis; BCD were all diagnosed as BDA3.

**Figure 2A** shows the normal left hand-wrist X-ray file without BDA3. The definition of BDA3 varies among different studies. Therefore, we chose a relatively objective definition (8), wherein BDA3 was considered when the middle phalanx of the fifth finger was shorter than half of the middle phalanx of the fourth finger (**Figure 2B**). All cases met this standard regardless of a curved middle phalanx of the fifth finger to the radial side (**Figure 2C**), or conical epiphysis (**Figure 2D**) were classified as the BDA3 group; whereas those who did not satisfy the criteria were classified as the no BDA3 group. We performed the x-ray for the purpose of diagnosing BDA3, and bone age was not analyzed in the study participants. If several left-hand wrist X-ray film were performed, we only selected their first X-ray film to analyze.

### Statistical Analysis

Statistical analysis was performed using SPSS version 22.0(IBM, NY, USA). Continuous variables were described as mean ± standard deviation (SD), and the differences between the BDA3 and no BDA3 groups were tested using the t-test. The categorical variables were described by frequency and percentage n (%), and the differences between different groups were tested using the *χ^2^* test. Statistical significance was set at *P* < 0.05. The effects of GH therapy were calculated based on the changes in children’s HtSDS (ΔHtSDS) during treatment.

## Results

A total of 752 children with short stature or normal short stature were included in this survey. We included a total of 463 cases of short stature children, wherein 279 had idiopathic short stature (ISS), 91 with growth hormone deficiency (GHD), 42 with Turner syndrome (TS), 27 with small for gestational age (SGA), and 24 had other conditions (including 11 cases of hypothyroidism, 8 cases of preterm infants, 3 cases of Noonan syndrome, 1 case of Laron syndrome, and 1 case of DiGeorge syndrome). The other 288 patients were in the normal short group (**Table 1**).

**Table 1:** The prevalence of BDA3 in different groups [n (%)].

	N	BDA3		*χ^2^*	*p*
		NO	YES		
Normal short	288	240 (83.3)	48 (16.7)	11.096	0.001
Short stature	463	337 (72.8)	126 (27.2)		
ISS	279	199 (71.3)	80 (28.7)	17.841	0.007
GHD	91	76 (83.5)	15 (16.5)		
TS	42	24 (57.1)	18 (42.9)		
SGA	27	16 (59.3)	11 (40.7)		
Others	24	22 (91.7)	2 (8.3)		

BDA3, brachydactyly type A3; ISS, idiopathic short stature; GHD, growth hormone deficiency; TS, Turner syndrome; SGA, small for gestational age.

**Figure 3:**
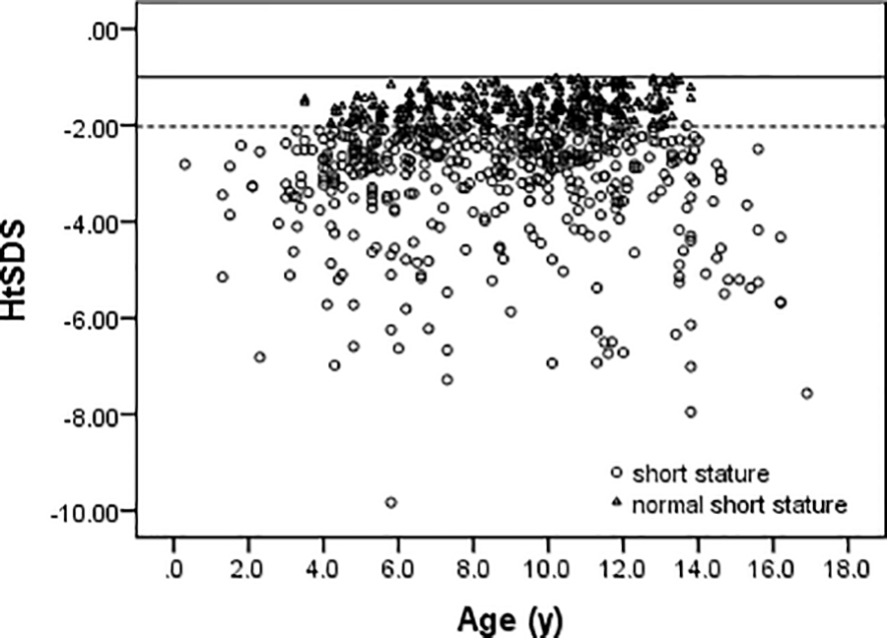
The age and HtSDS distribution of all cases.

Among 752 patients, there were 392 boys and 360 girls in this study. The overall ratio of boys to girls was 1.08:1. In case of the GHD, ISS, SGA, and normal short stature groups, the ratios of boys to girls were 2.92:1, 1.06:1, 1.05:1, and 0.93:1, respectively. The age and HtSDS distributions of all cases are shown in **Figure 3**. The mean age is 8.8 ± 3.1 years (range 0.3–16.9 years), and the HtSDS is -2.56 ± 1.24 (range -9.83 to -1.01).

### Prevalence of BDA3

The overall prevalence of BDA3 was 23.1% (174/752), with a higher prevalence in girls at 30.8% (111/360) compared to boys at 16.1% (63/392) (*P* < 0.01). Even excluded those girls with Turner syndrome, the prevalence of BDA3 in girls (29.2%) is still significantly higher than that in boys (16.1%) (*P* < 0.01).

We further analyzed the prevalence of BDA3 in the normal short stature and short stature groups with different etiologies. **Table 1** shows that the prevalence of BDA3 in the short stature group was 27.2%, which was significantly higher than that in the normal short group (16.7%). In the short stature group, the prevalence of BDA3 in patients with TS and SGA were exceed 40%. The prevalence of BDA3 in patients with GHD was lower than that in the normal short group. There was only one case of BDA3 in hypothyroidism and preterm patients. There were significant differences in the prevalence of BDA3 among the different groups.

### Comparison of Children’s Characteristics Between the BDA3 and No BDA3 Group

**Table 2** shows the general information, birth size, growth level, and mid-parental height of children in the BDA3 and no BDA3 groups. The chronological age and bone age of children in the BDA3 group were smaller than those in the no BDA3 group, but the difference between the chronological age and bone age (BAD) in these two groups was similar. This showed that BDA3 may not be related to the process of skeletal maturation and bone aging. Meanwhile, we observed that the birth weight, birth length, HtSDS, and WtSDS of the BDA3 group were slightly smaller than those in the no BDA3 group, but there were no significant differences (P > 0.05) (**Table 2**). In addition, the mid-parental height in the BDA3 group was also shorter than that in the no BDA3 group, which was significantly different in short stature children.

**Table 2:** The comparison of children’s characteristics between BDA3 and no BDA3 group (Mean ± SD).

	BDA3		*Difference (95%CI)*	*t*	*p*
	NO	YES			
Normal short group					
N	240	48			
Chronology age (y)	9.5 ± 2.5	8.9 ± 2.7	0.72 (-0.06,1.51)	1.813	0.071
Bone Age (y)	9.6 ± 2.6	8.5 ± 2.8	1.09 (0.02,2.16)	2.007	0.047
BAD (y)	0.2 ± 0.9	0.3 ± 0.9	-0.04 (-0.34,0.28)	-0.233	0.816
Birth Weight (kg)	3.14 ± 0.52	3.11 ± 0.46	0.03 (-0.14,0.19)	0.356	0.772
Birth Length (cm)	49.4 ± 2.1	48.9 ± 2.4	0.44 (-0.45,1.33)	0.983	0.327
HtSDS	-1.54 ± 0.26	-1.60 ± 0.29	0.05 (-0.03,0.14)	1.325	0.186
WtSDS	-1.00 ± 0.80	-1.15 ± 1.06	0.16 (-0.11,0.42)	1.151	0.251
Mid-Parent Height (cm)	164.0 ± 7.2	161.7 ± 7.3	2.27 (-0.06,4.60)	1.919	0.056
Short stature group					
N	337	126			
Chronology age (y)	8.5 ± 3.5	8.0 ± 3.4	0.48 (-0.21,1.19)	1.355	0.176
Bone Age (y)	7.8 ± 3.3	7.1 ± 3.4	0.76 (-1.33,1.64)	1.674	0.095
BAD (y)	1.4 ± 1.7	1.1 ± 1.4	0.26 (-0.11,0.63)	1.385	0.167
Birth Weight (kg)	3.06 ± 0.53	2.98 ± 0.48	0.08 (-0.04,0.19)	1.355	0.176
Birth Length (cm)	49.3 ± 2.1	48.8 ± 1.9	0.57 (-0.10,1.25)	1.672	0.096
HtSDS	-3.17 ± 1.23	-3.22 ± 1.11	0.05 (-0.20,0.30)	0.396	0.692
WtSDS	-1.99 ± 0.94	-2.08 ± 0.99	0.09 (-0.11,0.29)	0.897	0.370
Mid-Parent Height (cm)	163.6 ± 7.7	160.7 ± 7.2	2.9 (1.3,4.5)	3.607	0.000

BDA3, brachydactyly type A3; BAD, Chronology age- Bone Age; HtSDS, height standard deviation score; WtSDS, weight standard deviation score; Mid-Parent Height, (father height + mother height ±13cm)/2.

**Figure 4:**
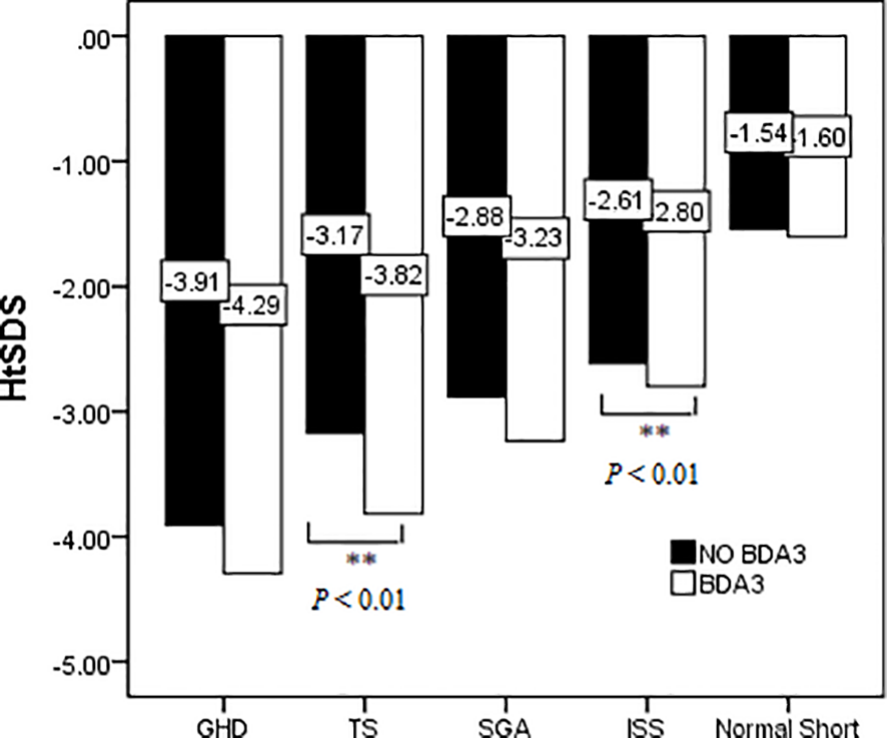
Effect of BDA3 in children’s HtSDS with different etiologies. ** means that there is a significant difference p < 0.01.

The short stature group consisted of patients with different etiologies; therefore, we further compared the HtSDS of children with different diagnoses to analyze whether patients with BDA3 were shorter than those without BDA3. **Figure 4** shows that in all short stature patients, there was a tendency of the HtSDS of children with BDA3 slightly lower than those without BDA3, but the differences were statistically significant only in TS and ISS patients. In patients with GHD and SGA, there were no significant differences in HtSDS between the BDA3 and no BDA3 groups.

### Association of BDA3 and the Therapeutic Effect of GH on Short Stature Children

Among the 463 short stature children in this survey, 346 received GH treatment for longer than one year. However, patients diagnosed with GHD, TS, SGA, and premature birth have confounding factors, such as growth hormone deficiency, chromosomal abnormality, and fetal abnormality, which render the analysis of the impact of BDA3 on the therapeutic effect of GH impossible. Therefore, we only analyzed whether BDA3 affected the therapeutic effect of GH in children with ISS without other confounding factors.

**Table 3** shows the age, height, and GH therapeutic effect of ISS patients with BDA3. ISS patients in the BDA3 group were younger and shorter than those in the no BDA3 group, but there was no significant difference in their HtSDS between these two groups. During the four years of GH therapy, the therapeutic effect (ΔHtSDS) decreased annually, and there were no significant differences in each year’s ΔHtSDS between the BDA3 and no BDA3 groups. At the end of treatment, there were still no significant differences in the duration of treatment and total ΔHtSDS between the two groups.

**Table 3:** The therapeutic effects of GH on ISS patients in BDA3 and no BDA3 group (mean ± SD).

	BDA3		*t*	*p*
	NO	YES		
Start of therapy				
N	153	55		
Age(y)	8.4 ± 3.3	8.0 ± 3.4	0.747	0.456
Height(cm)	118.2 ± 16.1	114.9 ± 17.4	1.290	0.199
Mid-Parent Height (cm)	162.7 ± 7.9	160.1 ± 8.0	1.990	0.048
HtSDS	-2.54 ± 0.56	-2.70 ± 0.69	1.690	0.092
GH dose(IU/kg/d)	015 ± 0.02	0.16 ± 0.02	-1.928	0.055
After 1st year of therapy				
N	147	48		
Height(cm)	127.0 ± 16.0	123.8 ± 17.7	1.174	0.242
HtSDS	-1.78 ± 0.59	-1.96 ± 0.81	1.669	0.097
ΔHtSDS_1st_	0.77 ± 0.39	0.79 ± 0.29	-0.292	0.771
After 2nd year of therapy				
N	96	34		
Height(cm)	135.0 ± 15.1	128.3 ± 15.3	2.053	0.042
HtSDS	-1.35 ± 0.69	-1.55 ± 0.89	1.371	0.173
ΔHtSDS_2nd_	0.45 ± 0.31	0.50 ± 0.31	-0.823	0.412
After 3rd year of therapy				
N	40	19		
Height(cm)	140.6 ± 12.6	128.1 ± 17.9	3.002	0.004
HtSDS	-1.00 ± 0.67	-1.30 ± 0.90	1.458	0.150
ΔHtSDS_3rd_	0.28 ± 0.22	0.20 ± 0.30	1.167	0.248
After 4th years of therapy				
N	23	8		
Height(cm)	144.7 ± 13.7	125.3 ± 3.7	3.928	0.001
HtSDS	-0.90 ± 0.59	-1.02 ± 0.52	0.507	0.616
ΔHtSDS_4th_	0.23 ± 0.34	0.10 ± 0.22	1.064	0.297
The end of therapy				
N	153	55		
Age(y)	11.1 ± 3.3	10.8 ± 3.3	0.511	0.610
GH therapy time(m)	28.5 ± 16.4	29.3 ± 19.2	-0.293	0.770
Height(cm)	138.3 ± 17.4	135.6 ± 15.6	1.019	0.310
HtSDS	-1.25 ± 0.76	-1.39 ± 0.77	1.168	0.244
ΔHtSDS _total_	1.29 ± 0.69	1.31 ± 0.68	-0.212	0.832

Mid-Parent Height, (father height + mother height ±13cm)/2; HtSDS, height standard deviation score; ΔHtSDS, the changes in children’s HtSDS during treatment.

## Discussions

We analyzed the association of BDA3 with short stature in a pediatric population. We also investigated the prevalence of BDA3, response to GH therapy in short stature children with and without BDA3, and characteristic differences between children with and without BDA3. The prevalence of BDA3 in short stature children is higher than that in normal height children, with a female predominance. BDA3 did not affect the effect of GH therapy on children with BDA3.

Because left-hand wrist radiography carries a risk of radioactive exposure, it is difficult to perform large-scale surveys using this modality in the general population, so there are almost no studies on the prevalence of BDA3 in China. In this survey, we found that the total prevalence of BDA3 in short stature and normal short children was 23.1%, which is significantly higher than the 6.95% in a relatively normal population survey in China (8), and was also higher than that of Chinese descents in the United States (12.5%) and a previous small sample survey in China conducted in 1967 (5.0%) (9, 10). Meanwhile, the prevalence of BDA3 in the short stature group was 27.2%, which was also significantly higher than that in the normal short group (16.7%). These results revealed that BDA3 is more commonly seen in children with short stature. In addition, BDA3 is more commonly seen in girls than in boys, which is consistent with the conclusions of other Asian populations (11). In a series of surveys on the Japanese population from 1942 to 1973 and one survey on the Japanese descents in the United States, a female predominance was likewise noted (2, 12). In other surveys in Native Americans, Mexico, and Pacific island countries, BDA3 is also more frequently seen in females (12–14). However, this phenomenon was not observed in Caucasians (15). The reason why BDA3 is more common in short children and girls needs to be explained by its exact mechanism.

The mechanism and pathogenic gene of BDA3 remain unclear. This may be a combination of complicated mechanisms involving multiple genes and pathogenic pathways. In recent years, it has been found that BDA3 may be an autosomal dominant condition with an obvious familial genetic tendency (1). A Chinese study in 2020 stated that the deletion of the *HOXD13* gene is related to the occurrence of familial BDA3 and BDA4 (16). Vasques et al. reported that BDA3 was observed in 64.3% of hand radiographs from individuals heterozygous from *IHH* variants initially classified as ISS (17). However, Williams et al. did not support the autosomal dominant inheritance model of BDA3 (2). In this study, we found that the prevalence of BDA3 in children with chromosomal abnormalities such as TS was 42.9%, suggesting that the deletion of specific spots on the X chromosome may be related to the occurrence of BDA3. Otherwise, BDA3 may occur in the fetus and is related to the abnormal development of the fetus during the first nine weeks of pregnancy (18), which directly affects the birth weight and body length of the fetus. The prevalence of more than 40% of BDA3 in SGA children in this study also indirectly confirms the above conclusion. However, some children with BDA3 grow normally during fetal development (delivered with a normal birth length according to standardized growth charts) but later exhibit short stature as they age, as seen in the patients with ISS. Therefore, the prevalence of BDA3 in children with ISS is also higher than in normal children, and this often results in a shorter adult height (18). In addition, BDA3 can appear in some syndromes such as Silver Russell syndrome, Coffin-Siris syndrome, Down syndrome (5, 19), and a pair of identical twin girls (8). Further research needs to confirm whether the specific pathogenesis of BDA3 involves autosomal, sex chromosomes, or multiple etiologies. We only know that the prevalence of BDA3 in GHD patients is relatively low, even lower than that in the normal short group, which may imply that the occurrence of BDA3 is independent of GH secretion.

BDA3 did not affect the therapeutic effect of GH on short-stature children. **Table 3** shows no significant differences in ΔHtSDS between the BDA3 and no BDA3 groups during the first, second, third, and fourth years of GH therapy. At the end of the therapy, there were no differences in HtSDS and total ΔHtSDS between the two groups. This further confirmed that BDA3 has nothing to do with the GH pathway, and short stature children with simple BDA3 can also improve their height by GH therapy similar to those without BDA3. Pereda and Vasques have confirmed that GH therapy can improve the height of children with short stature with other types of brachydactyly, but their study had no normal control group (6, 17, 20). Besides, our study suggests that BDA3 was not related to the process of skeletal maturation, bone aging, and the degree of short stature, which may be due to the relatively small sample of children in this study. So further longitude study is still needed to explore the relationship between BDA3, skeletal maturation process, and adult short stature.

So far, this is the first study on the effect of BDA3 on the therapeutic effects of GH in short stature children. The strength of this study is the longitudinal follow-up data of GH therapy on short stature children with BDA3. However, our study also has some limitations. First, in this study, medical records from July 2009 to July 2021 were retrospectively analyzed. Before 2015, the clinical application of second-generation sequencing technology was very limited. Therefore, some short stature children with bone abnormalities or bone genetic disorder were not definitely diagnosed with these conditions. After 2015, almost all short stature children with multiple skeletal deformities were evaluated by a geneticist. However, in this study, we only included children with isolated brachydactyly type A3 (BDA3) and not those with syndromic forms of BDA3. Second, cross-sectional data were collected from a single pediatric clinic, so the sample is relatively small and cannot represent all kinds of short-stature children. Therefore, whether BDA3 can aggravate short stature in children still needs to be verified using larger sample surveys. Third, this study did not include genetic characteristics and whole family spectrum analyses of BDA3 patients, so we cannot distinguish whether BDA3 is hereditary or spontaneous, and weather different kind of BDA3 has different effect on children’s height growth and their therapeutic effect of GH.

## Conclusions

In conclusion, BDA3 is more commonly seen in short stature children than in normal height populations and manifests predominantly in girls. BDA3 occurrence is independent of the GH pathway and does not affect the therapeutic effect of GH on short-stature children. However, the mechanisms and pathogenic genes of BDA3 are not clear. Therefore, further research should include genetic characteristics and whole family spectrum analyses of BDA3 patients to explore the inheritance and pathogenesis mechanism of this disease.

## Data Availability Statement

The raw data supporting the conclusions of this article will be made available by the authors, without undue reservation.

## Ethics Statement

The studies involving human participants were reviewed and approved by Ethical Review Committee of the Capital Institute of Pediatrics. Written informed consent to participate in this study was provided by the participants’ legal guardian/next of kin.

## Author Contributions

HW was involved in data collecting and review, in charge of this paper’s results interpretation and paper writing. HL was in charge of survey design, data review supervision, results interpretation, and paper writing. YL was involved in data collecting and review. All authors agreed with the data interpretation and approved the final version of the manuscript.

## Funding

This study was supported by The Special Fund of the Pediatric Medical Coordinated Development Center of Beijing Hospitals Authority (XTZD20180403), Public service development and reform pilot project of Beijing Medical Research Institute (BMR2019-11), and CAMS Innovation Fund for Medical Sciences (CIFMS) (2016-I2M-1-008).

## Conflict of Interest

The authors declare that the research was conducted in the absence of any commercial or financial relationships that could be construed as a potential conflict of interest.

## Publisher’s Note

All claims expressed in this article are solely those of the authors and do not necessarily represent those of their affiliated organizations, or those of the publisher, the editors and the reviewers. Any product that may be evaluated in this article, or claim that may be made by its manufacturer, is not guaranteed or endorsed by the publisher.
